# Therapeutics and Materia Medica

**Published:** 1864-11

**Authors:** 


THERAPEUTICS AND MATERIA MEDICA. A Systematic Treatise on the Action and Uses of Medicinal Agents, including their description and history. By ALFRED STILTE, M.D., Professor of Theory and Practice of Medicine in the University of Pennsylvania; Physician to St. Joseph's Hospital, &c., &c., &c. Second Edition, revised and enlarged. In two volumes. Philadelphia: BLANCHARD & LEA. 1864.

This is a full and valuable treatise on Materia Medica, consisting of two volumes of about 800 pages each. The first edition has been long enough before the profession to have its merits well known; and it has justly taken rank among the best standard works on the subject of which it treats.

For sale by W. B. Keen & Co., 148 Lake Street, Chicago.

